# Dynamic Three-Dimensional Shoulder Mri during Active Motion for Investigation of Rotator Cuff Diseases

**DOI:** 10.1371/journal.pone.0158563

**Published:** 2016-07-19

**Authors:** Christine Tempelaere, Jérome Pierrart, Marie-Martine Lefèvre-Colau, Valérie Vuillemin, Charles-André Cuénod, Ulrich Hansen, Olivier Mir, Wafa Skalli, Thomas Gregory

**Affiliations:** 1 Laboratory of Biomechanics, Arts et métiers ParisTech, Paris, France; 2 Upper Limb Surgery, European Hospital Georges Pompidou, APHP, Université Paris Descartes, Sorbonne Paris Cité, Faculté de Médecine, Paris, France; 3 Physical Medicine and Rehabilitation Unit, Cochin Hospital, APHP, Université Paris Descartes, Sorbonne Paris Cité, Faculté de Médecine, Paris, France; 4 Radiology Unit, European Hospital Georges Pompidou, APHP, Université Paris Descartes, Sorbonne Paris Cité, Faculté de Médecine, Paris, France; 5 Department of Mechanical Engineering, Imperial College London, London, United Kingdom; 6 Institut MOVEO, Université Paris Descartes, Sorbonne Paris Cité, Faculté de Médecine, Paris, France; Mathematical Institute, HUNGARY

## Abstract

**Background:**

MRI is the standard methodology in diagnosis of rotator cuff diseases. However, many patients continue to have pain despite treatment, and MRI of a static unloaded shoulder seems insufficient for best diagnosis and treatment. This study evaluated if Dynamic MRI provides novel kinematic data that can be used to improve the understanding, diagnosis and best treatment of rotator cuff diseases.

**Methods:**

Dynamic MRI provided real-time 3D image series and was used to measure changes in the width of subacromial space, superior-inferior translation and anterior-posterior translation of the humeral head relative to the glenoid during active abduction. These measures were investigated for consistency with the rotator cuff diseases classifications from standard MRI.

**Results:**

The study included: 4 shoulders with massive rotator cuff tears, 5 shoulders with an isolated full-thickness *supraspinatus* tear, 5 shoulders with tendinopathy and 6 normal shoulders. A change in the width of subacromial space greater than 4mm differentiated between rotator cuff diseases with tendon tears (massive cuff tears and *supraspinatus* tear) and without tears (tendinopathy) (p = 0.012). The range of the superior-inferior translation was higher in the massive cuff tears group (6.4mm) than in normals (3.4mm) (p = 0.02). The range of the anterior-posterior translation was higher in the massive cuff tears (9.2 mm) and *supraspinatus* tear (9.3 mm) shoulders compared to normals (3.5mm) and tendinopathy (4.8mm) shoulders (p = 0.05).

**Conclusion:**

The Dynamic MRI enabled a novel measure; ‘Looseness’, i.e. the translation of the humeral head on the glenoid during an abduction cycle. Looseness was better able at differentiating different forms of rotator cuff disease than a simple static measure of relative glenohumeral position.

## Introduction

Musculoskeletal diseases of the shoulder are frequent, rotator cuff diseases alone [1] affecting up to 30% of the population. Currently, MRI with its ability to evaluate the soft tissues of the rotator cuff is the standard imaging technique to aid the detection and best treatment of rotator cuff diseases. Still, half of all rotator cuff diseases patients have persistent pain despite 12 to 18 months of treatment [2] and it seems rotator cuff diseases are still poorly understood.

Shoulder motion is the result of the synergy and combined movement of the scapula-humeral and the scapula-thoracic joints. The rotator cuff muscles are key activators for the control of this motion and rotator cuff diseases are associated with alteration of this motion [3]. The suggestion of this work is that a technique which could assess the changed kinematics due to rotator cuff diseases, as well as a simultaneous assessment of the loaded soft tissues of the rotator cuff would provide an improved ability to detect rotator cuff diseases as well as an aid to decide on the best treatment.

Cadaver studies of shoulder kinematics inherently provide limited information about the complex muscle activation pattern of the shoulder during active arm elevation [4]. *In vivo* methods based on external markers are questionable because of the movement between the skin and the bone structures during shoulder motion and the difficulty of interpreting EMG signals from deep muscles [5,6]. Conventional X-rays [7–9], low dose Stereography System™and EOS [10] as well as bi-planar fluoroscopy [11–13] have all been used to analyze the humeral head translation relative to the glenoid. However, these methods are all limited by the involved radiation exposure as well as the inability to assess the status of the soft tissues of the rotator cuff.

Ultrasound scanning [14] has the ability to assess both the kinematics of joint movement and the status of the soft tissues but has the disadvantage of being operator dependent. In-vivo three-dimensional (3D) MRI techniques [3, 15–22] have investigated shoulder kinematics by simulating the physiological movement as a series of static positions and performing an MRI scan at each position. The shoulder muscles were loaded in an attempt to produce physiological kinematics but the loading was isometric due to the static position. The authors recognized that isotonic muscle loading and continuous dynamic movement of the arm may produce more realistic results. The scan time at each position was 4 min and it may also have been difficult for the patient to maintain a consistent level of muscle activity for this length of time. *In vivo* two-dimensional (2D) MRI techniques have allowed investigations during active and continuous arm abduction [22]. However, these techniques rely on a predefined abduction plane and can only analyze movement within this 2D plane. The difficulty for the patient to adhere to the predefined abduction plane is an additional limitation of this method.

Recently, Pierrart et al. [23] established a real time 3D MRI technique (Dynamic-MRI) that enabled the noninvasive monitoring of the kinematics of the shoulder complex during slow active arm elevation. The 3D nature of the Dynamic-MRI avoided the problems associated the 2D MRI techniques mentioned above. The acquisition process of the Dynamic-MRI technique was fast enough to carry out multiple scans while the patient abducted the shoulder in a continuous motion. The resulting kinematics was that of an isotonically and naturally loaded shoulder joint [23]. While the fast acquisition resulted in a quality of the images insufficient for assessment of the loaded soft tissues it seems likely that further development will lead to better quality images.

Pierrart et al. evaluated the Dynamic-MRI technique only in normal shoulders. The objective of this paper was to evaluate if Dynamic MRI provides novel kinematic data that can be used to improve the understanding, diagnosis and best treatment of rotator cuff diseases.

## Methods

### Patient population and selection

Patients presenting with pre-existing MRI scans showing cuff tear disease were selected for the study on a consecutive basis, from June to December 2014. In all cases but one (patient 7), the physiopathology of rotator cuff diseases was degenerative. In patient 7, the onset was traumatic. Patients with a past history of surgery or algodystrophia were excluded. Each participant was given an informed consent form to read and sign and the local ethics committee (CPP Paris-Ile-De-France 2) approved all parts of this study.

Global function was classified according to the Constant score [24] and the active forward flexion and abduction were measured.

The quality of the Dynamic-MRI scans were not adequate for assessing the rotator cuff but appropriate for tracking the bone. Therefore, prior to Dynamic-MRI, the status of the rotator cuff was assessed from standard shoulder MRI scans by a radiologist with expertise in musculoskeletal diseases (VV) and the shoulders divided into 4 grades of cuff disease: normals (group N), tendinopathy in the *supraspinatus* tendon but not involving any full-thickness tears (group tendinopathy), isolated full-thickness *supraspinatus* tear (group SST) and massive rotator cuff tear involving at least two tendons. In cases of full thickness tears, the tendon retraction on the frontal plane was classified according to Patte et al. [25]. For all groups and all rotator cuff tendons except for the *teres minor*, two additional parameters were assessed: muscle atrophy, scored using the three stage classification of Thomazeau et al. [26, 27] and the muscle fatty degeneration, scored according to the five stages classifications of Goutallier et al. [28].

### 3D Dynamic-MRI technique

A dynamic sequence was performed for each shoulder. The protocol was described by Pierrart et al. [23]: the first acquisition was a Fiesta 3D dynamic sequence then a sequence at rest was performed. Prior to imaging, the procedure was explained to the patients, specially the requested motion (pace, orientation of the elbow, position in the scanner and different sequences). Patients rehearsed the desired abduction motion once outside the MRI scanner, and once inside the MRI scanner. Each patient was placed in lateral decubitus, allowing the scapula to tilt, within a closed-bore MRI scanner (Sigma 1.5 Tesla system, General Electric Milwaukee,WI) including a shoulder coil. The patient’s arm was positioned unrestrained along the body with the elbow flexed 90° and the hand placed on the abdomen. This position defined the reference position. During the dynamic sequence, the pace of the shoulder motion was maintained by counting from 0 to 28, corresponding to 28 seconds; consequently it was easy for the patient to understand when to start and how fast to move the arm. During abduction the elbow would abut against the inner wall of the scanner limiting the range of motion. The degree of humero-thoracic active abduction when this happened varied from shoulder to shoulder depending on patient-specific factors such as weight and size but was on average 38 (20–56, 8)°, 52 (42–62,3)°, 49 (40,2–67)° and 43(30–60)°, for the massive cuff tears *supraspinatus* tear, tendinopathy and control groups, respectively. The exception was patient 6 whom had limited abduction (20°) also outside the MRI.

Through the MRI scanner window, the following parameters of the patient motion were monitored and readjusted if needed: direction (abduction in the plane of the scapula), pace (maximal abduction in 28 seconds) and uninterrupted motion. The sequence of 28 seconds was repeated two or three times to obtain an optimal fiesta sequence. Subsequently, a standard shoulder MRI including 4 sequences (T1 coronal, T1 sagittal, T1 axial, and T2 sagittal FATSAT) was performed at rest. For each patient, the overall examination time, including the time to explain, read and sign the consent form and time to get in and out the MRI, lasted 20 to 25 minutes.

### 3D reconstruction and registration

The following steps were performed blinded to the rotator cuff diseases groups. Commercial medical imaging software (AVIZO, Visualisation Science Group,VSG; Burlington,MA) was used to reconstruct 3D shoulder models from coronal T1 sequences as previously described [23]. Using the Fiesta sequence, 8 shoulders reconstructions, corresponding to 8 successive positions during abduction, were obtained. Using best-fit alignment another software package (Geomagic, Morrisville, NC) semi-automatically registered these models to the reference model (0° abduction). From the reconstructed models, the position of anatomical areas (the humeral head, shaft and greater tuberosity of the humerus, acromion process, and the glenoid of the scapula) were determined.

MathLab (MathWorksVR, software) was used to compute, animate, and analyze the kinematics of the gleno-humeral joint as follows: A mean least squares ellipse was fitted onto the contour of the glenoid region and subsequently the glenoid coordinate system was characterized: minor axis as the anterior-posterior axis (X), major axis as the superior-inferior axis (Y) and orthogonal to the X-axis, and the Z-axis as the cross product of the X- and Y-axes. The center of the ellipse was used as the center of the glenoid coordinate system and X- and Y- axes defined the glenoid plane. For determining the central point of the humeral head, a sphere was fitted to the humeral surface of the humeral head. This central point was projected perpendicularly onto the glenoid plane and its location was defined in the glenoid coordinate system. Glenohumeral abduction was defined as the angle formed by the longitudinal axis of the humerus and the glenoid plane.

The width of the subacromial space was defined as the shortest distance between the superior aspect of the proximal humerus contour (humeral head or greater tuberosity) and the inferior aspect of the acromion. The translation of the humeral head on the glenoid was defined as the movements in the X- and Y-directions (roughly anterior-posterior and superior-inferior directions, respectively) on the glenoid plane of the projected of center of the humeral head.

The intra-observer reproducibility was tested for one intermediate position of one normal shoulder. The 3D reconstruction of the same intermediate position was repeated 6 times, each reconstruction followed by the registration step. The difference between the largest values was calculated for three parameters: coordinates on the X and Y-axes, width of the subacromial space and measure of the gleno-humeral abduction.

### Statistical analysis

Statistical analysis software GraphPad Prism 5.00 (GraphPad software, San Diego, California) was used to determine relationships between variables. Significance was set at p < 0.05. In order to compare quantitative variables of two groups (independent variables), a Student parametric test was used when the variables had a normal distribution, and a Mann-Withney test was used if the distribution was not normal. To compare quantitative variables of more than two groups, a Kruskal-Wallis test was used. The post-hoc analysis, which compares a group with another, was performed with the Dunn Test.

## Results

This prospective study involved 14 shoulders from 11 patients with rotator cuff diseases (mean age 67, range: 53–79) years, 10 females, 4 right shoulders) and a control group of 6 normal shoulders from 4 volunteers (mean age 34.2 (30–45) years, 3 females, 4 right shoulders). Table 1 depicts the clinical evaluation of patients. No patient had a past history of shoulder instability. Patients Body mass index remained between 20 and 25 kg/m^2^ (Table 1).

**Table 1:** Description of clinical evaluation of patients and healthy volunteers.

**Patient**	**Shoulder specimen**	**Sex**	**Age (years)**	**Shoulder**	**Height (cm)**	**Weight (kg)**	flexion/abduction/external rotation (°)	**Constant score (/100)**
1*	N1	Male	30	right	175	60	180/120/80	92
1*	N2	Male	30	left	175	60	180/130/80	95
2*	N3	Female	35	right	160	45	180/120/70	97
2*	N4	Female	45	right	162	52	180/130/80	93
3*	N5	Female	33	right	161	54	180/110/80	92
4*	N6	Female	33	left	161	54	180/120/80	95
5	MCT1	Male	81	right	170	81	180/90/90	65
5	MCT2	Male	81	left	170	81	100/60/40	63
6	MCT3	Female	59	right	170	88	150/70/70	65
7	MCT4	Female	76	right	168	62	180/80/90	64
8	SST1	Female	63	right	157	64	180/80/90	75
9	SST2	Female	65	left	153	55	160/90/80	73
10	SST3	Female	68	right	150	65	180/85/70	76
7	SST4	Female	76	left	168	67	180/75/80	76
11	SST5	Female	71	right	155	60	180/50/70	77
12	TA1	Female	73	right	160	70	180/90/90	82
13	TA2	Female	53	right	158	68	160/90/80	74
14	TA3	Female	66	right	158	54	140/90/70	83
6	TA4	Female	59	left	170	88	100/40/30	75
15	TA5	Female	65	right	165	70	180/90/90	84

*: healthy subjects

Abbreviations: N, normal; MCT, massive rotator cuff tear; SST, supraspinatus tear; TA, tendinopathy.

Muscle atrophy, scored using the three stage classification of Thomazeau et al. [26, 27] and the muscle fatty degeneration, scored according to the five stages classifications of Goutallier et al. [28]. The result of this assessment for the massive cuff tears, *supraspinatus* tear and tendinopathy groups is shown in Table 2. The *teres minor* in all specimens of the massive cuff tears group was hypertrophic, but showed normal trophicity in the *supraspinatus* tear and tendinopathy groups. In the control group of normal shoulders, no tears or muscle atrophy or degeneration was observed.

**Table 2:** Assessment of rotator cuff.

Shoulder specimen	Extent of tendon retraction(Patte^24^)			Level of muscle atrophy(Thomazeau^26, 27^)			Level of fatty degeneration(Goutallier et al^28^)		
	*Subscapularis*	*Supra-spinatus*	*Infra-spinatus*	*Subscapularis*	*Supra-spinatus*	*Infra-spinatus*	*Subscapularis*	*Supra-spinatus*	*Infra-spinatus*
MCT1	I	III	III	II	III	III	II	III	IV
MCT2	I	III	III	III	III	III	IV	III	IV
MCT3	-	III	III	-	III	III	-	IV	IV
MCT4	I	III	III	III	III	III	IV	IV	IV
SST1	-	I	PT	-	-	-	-	-	-
SST2	-	I	-	-	-	-	-	-	-
SST3	-	I	PT	-	-	-	-	-	-
SST4	-	I	PT	-	II	II	-	II	II
SST5	-	I	-	-	II	II	-	II	II
TA1	-	-	-	-	-	-	-	-	-
TA2	-	PT	-	-	-	-	-	-	-
TA3	-	PT	-	-	-	-	-	-	-
TA4	-	-	-	-	-	-	-	-	-
TA5	-	-	-	-	-	-	-	-	-

Abbreviations: N, normal; MCT, massive rotator cuff tear; SST, supraspinatus tear; TA, tendinopathy; PT: partial tear; ‘-‘, normal.

**Fig 1:**
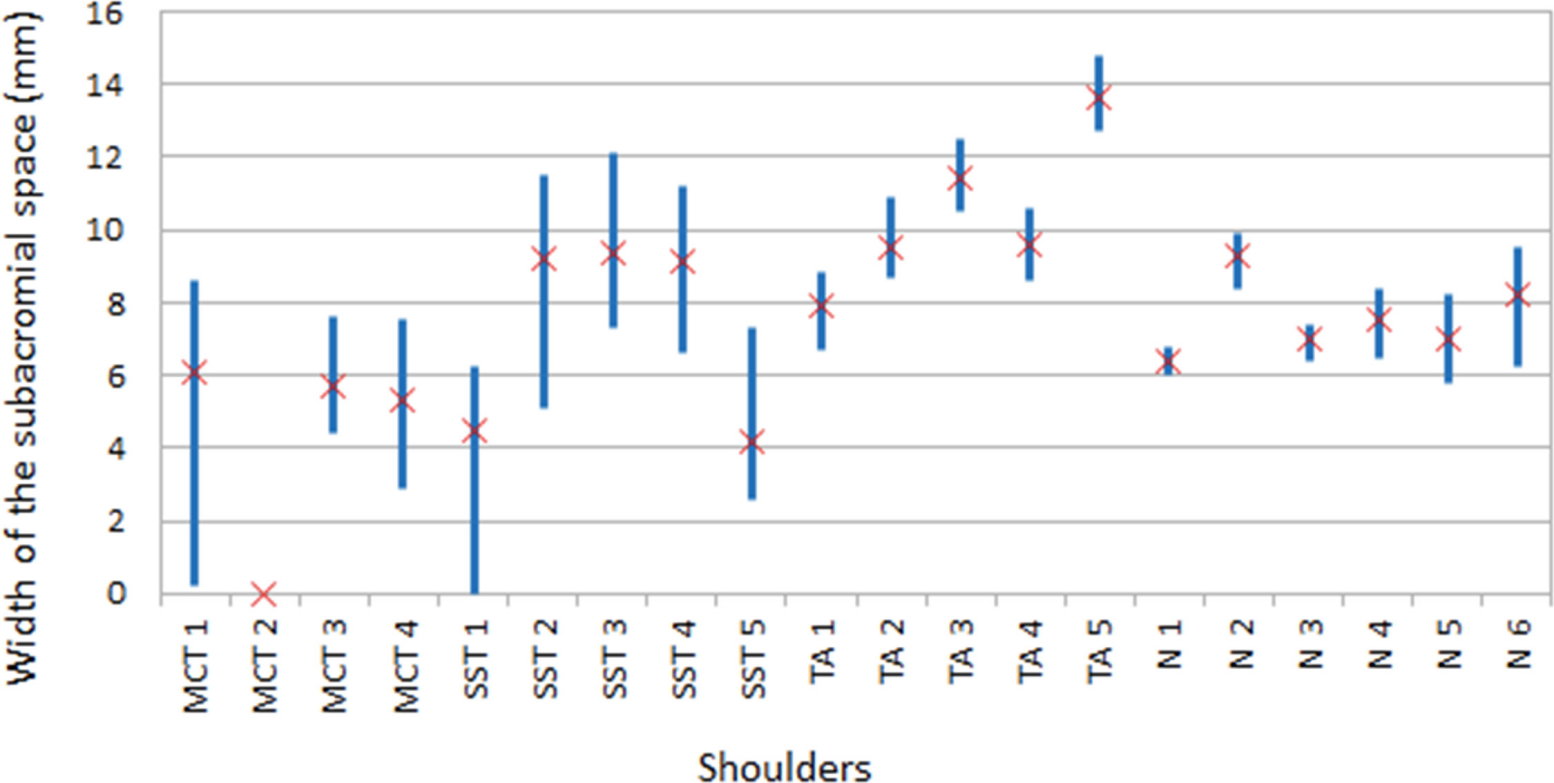
The width of the subacromial space during abduction motion.

The width of the subacromial space was recorded at 4 sec intervals throughout the 28 sec abduction cycle, thus producing 7 measurements for each shoulder. The average, WSS_avg_, and range, WSS_range_, of these 7 measurements for each specimen are shown in Fig 1. The mean of WSS_avg_ and WSS_range_ of each of the rotator cuff diseases groups are presented in Table 3. WSS_avg_ was lower in the massive cuff tears group than in any other group (p = 0.012) while a WSS_range_ of more than 4 mm differentiated control and tendinopathy shoulders from *supraspinatus* tear and massive cuff tears shoulder, i.e. between rotator cuff disease with and without tendon tears (p = 0.012).

**Table 3:** Mean of kinematic measures for each rotator cuff disease group

Group	WSS_avg(mm)_	WSS_range(mm)_	Y_avg(mm)_	Y_range(mm)_	X_avg(mm)_	X_range(mm)_
MCT	4.3	4.0	3.8	6.4	-0.8	9.2
SST	7.3	5.3	2.0	5.0	0.9	9.3
TA	10.4	2.1	0.3	4.4	-1.6	4.8
N	7.7	1.9	1.0	3.4	1.1	3.5

Abbreviations: N, normal; MCT, massive rotator cuff tear; SST, supraspinatus tear; TA, tendinopathy.

**Fig 2:**
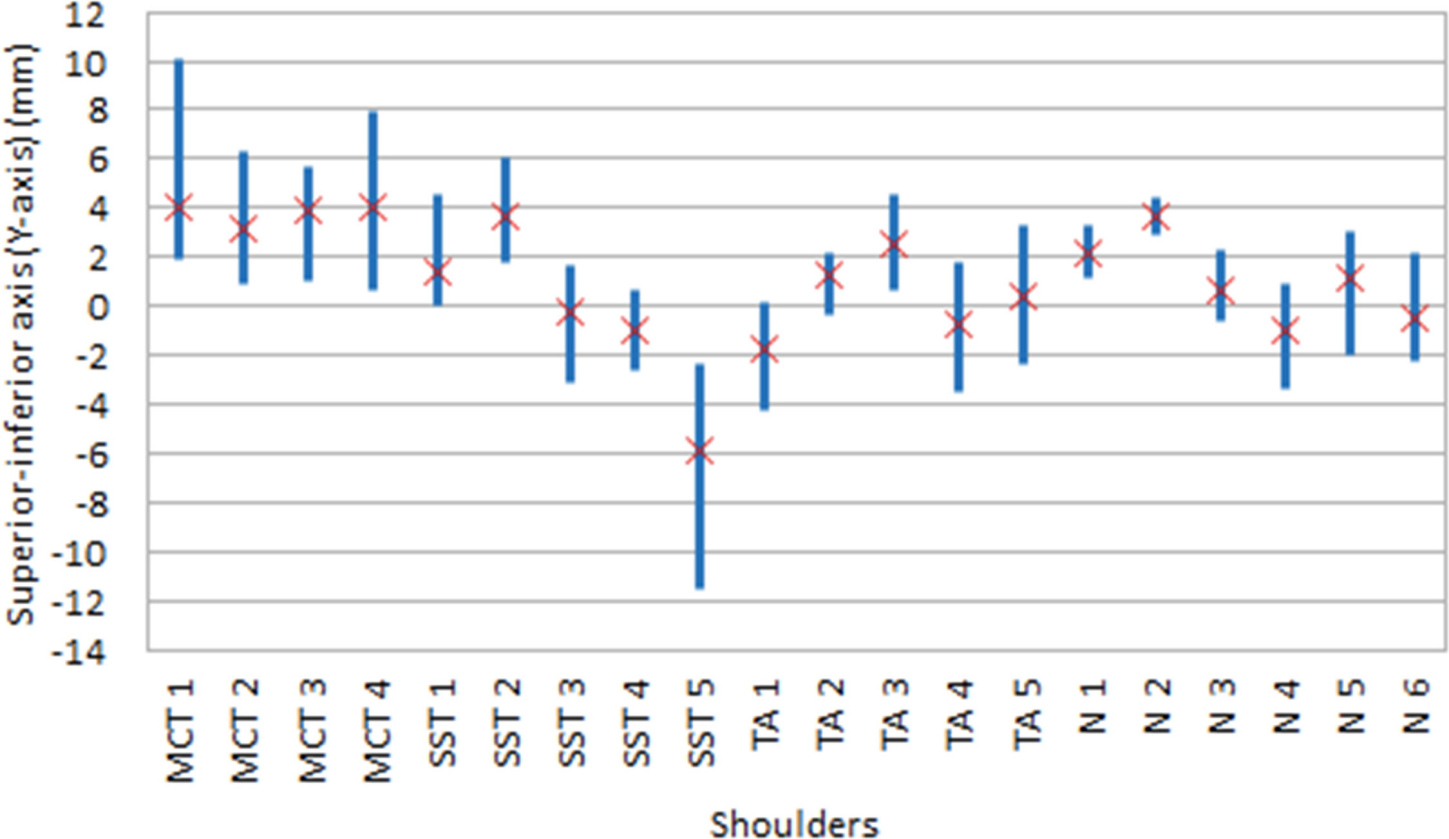
Translation of the humeral head along the Y-axis (superior-inferior direction) of glenoid coordinate system

**Fig 3:**
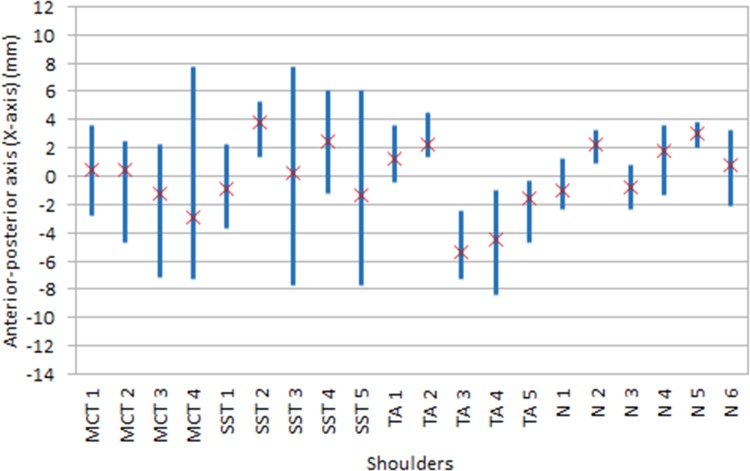
Translation of the humeral head along the X-axis (anterior-posterior direction) of glenoid coordinate system

Corresponding to the above description, the average and range of translation of the humeral head in the approximately superior-inferior direction (Y-direction) and anterior-posterior direction (X-direction) for each shoulder were termed Y_avg_ and Y_range_, and X_avg_ and X_range_, respectively. The Y_avg_ and Y_range_ for each specimen are shown in Fig 2 while X_avg_ and X_range_ are shown in Fig 3. Essentially, Y_avg_ and X_avg_ describes where the humeral head is located for most of the time during the abduction movement while Y_range_ and and X_range_ describes how much the humeral head ‘wobbles’ around during the abduction movement. The mean of Y_avg_, Y_range_, X_avg_, X_range_ of each of the specimen groups are presented in Table 3.

Y_range_ was higher in massive cuff tears shoulders compared to the control group (6.4 vs. 3.4 mm, p = 0.02), but not when compared to the tendinopathy (p = 0.11) and *supraspinatus* tear groups (p = 0.28). Finally, X_range_ was higher in massive cuff tears and *supraspinatus* tear shoulders when compared to the control and tendinopathy groups (9.2 and 9.3 vs. 4.8 and 3.5 mm, p = 0.05) (Table 3).

With respect intra-observer reproducibility test, the difference between extreme values was 2 mm in the X-direction, 1.9 mm in Y-direction, 1.3 mm for the width of the subacromial space, and 1.3° for the measure of the gleno-humeral abduction.

**Fig 4:**
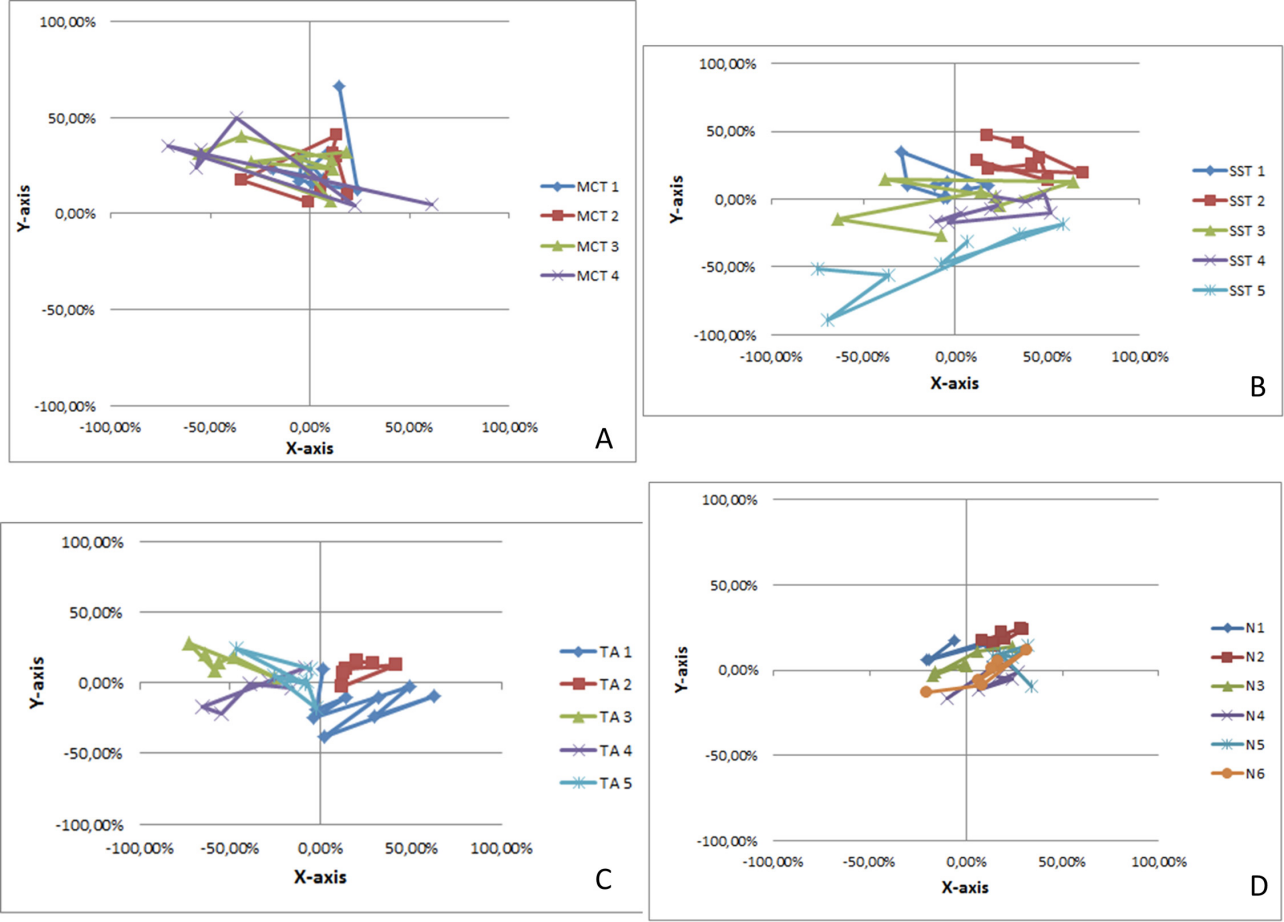
Monitoring of the humeral head center projection on to the glenoid for each of the 14 shoulders during abduction. The size of the glenoids was standardized so that each fit with a circle of 200% diameter (-100% to +100%). The locations of the humeral head center projections on to the glenoid are expressed in percentile. Fig 4A, massive rotator cuff tear; Fig 4B, *Supraspinatus* tear; Fig 4C, Tendinopathy alone; Fig 4D, normal shoulders.

Fig 4 summarize the monitoring of the humeral head center projection on to the glenoid for each of the 14 shoulders during abduction, for respectively massive rotator cuff tear (Fig 4A), *supraspinatus* tear (Fig 4B), tendinopathy alone (Fig 4C) and normal shoulders (Fig 4D).

## Discussion

The objective of this paper was to evaluate if Dynamic MRI provides novel kinematic data that can be used to improve the understanding, diagnosis and best treatment of rotator cuff diseases. The long-term aim of this work is to develop the Dynamic-MRI technique as a tool that can provide kinematic data and simultaneous MRI assessment of the loaded soft tissues of the rotator cuff during arm movement.

The most important finding of this work was that the Dynamic-MRI technique enabled a novel measure; ‘Looseness’, i.e. the translation of the humeral head on the glenoid during an abduction cycle (X_range_ and Y_range_). Looseness was better able at differentiating rotator cuff disease than a simple static measure of relative gleno-humeral position.

This study found that the subacromial space narrowed with rotator cuff disease (Fig 1 and Table 3) which is consistent with other studies [17, 29]. The reported width of the subacromial space varies between studies (5 to 9 mm for healthy shoulders [11, 17, 29]) and are not inconsistent with the values found here (Table 3).

The finding that superior-inferior excursion in torn shoulders is larger than in normals (Y_range_, Table 3) is related to the narrowing of the subacromial space and seems sensible considering the superiorly directed deltoid force and the reduced ability of the rotator cuff to compress and stabilise the joint. While this finding was not surprising it does provide some confidence in the Dynamic-MRI kinematic measurements. In contrast, the notable anterior-posterior excursion (Fig 3 and Table 3) was not expected. Most studies investigating the effect of rotator cuff disease have not considered the anterior-posterior gleno-humeral translation. However Bey et al. [11] did report that the anterior-posterior excursion was not restored (was larger than normal) by rotator cuff repair. To our knowledge, this study is the first to report that the range of anterior posterior motion (X_range_) increases significantly with rotator cuff diseases.

In fact, the superior-inferior motion (Y_range_) was less affected than anterior-posterior translation (Table 3) which may seem surprising. However, Bey et al. [11] showed that the superior-inferior excursion was restored by rotator cuff repair whereas anterio-posterior translation was not, which also indicate that anterior-posterior motion may be more affected by rotator cuff diseases. Previous studies [30, 31] have demonstrated that the glenoid is “flatter” in the anterior-posterior direction than in the superior-inferior direction and joint excursion in the anterior-posterior direction may depend more on the stability provided by the rotator cuff and, consequently, may be more affected by rotator cuff disease. Therefore analysis and improved understanding of excursion in the anterior-posterior direction may be critical to diagnosis and treatment of different conditions of rotator cuff diseases.

The dynamic MRI technique also allowed us to measure the ranges of the variables (WSS_range_, Y_range_, X_range_) during the motion; effectively how loose or unstable the joint was. This looseness was shown to be a better measure for identifying and specifying cuff tear disease than the average measures used in static techniques.

Although an analysis of the subacromial space combined with an analysis of the translations of the humeral head allowed identification of cuff tears and the degree of cuff tear pathology, statistical significance was not consistently found between tendinopathy shoulders and healthy shoulders. This may be due to the relatively few specimens in the study, which may have prevented statistically significant differences to be found for both analyses.

The abduction cycle was prescribed to take 28 seconds, which clearly does not represent physiological arm motion. However, image noise is inversely proportional to the acquisition speed and an acquisition phase lasting for 4 seconds, resulting in 7 acquisitions during the abduction motion, was chosen as a balance between image quality and realistic abduction times. Consequently, the MRI dynamic technique enabled kinematics analysis of the bone structures but not simultaneous visualization of the tendons. Visualization of the soft structures is a priority for future work.

Another limitation was the use of a closed-bore scanner. This scanner was chosen because of its common availability but resulted in the elbow abutting against the wall of the scanner during the motion, thus restricting humero-thoracic abduction to approximately 45°. However, the protocol could be easily transferred to an open-bore MRI scanner that would provide, in upright position, the full range of shoulder abduction.

This study did assess the intra-observer reproducibility of the technique. Considering all criteria (data on X-axis and Y-axis of the humeral head projection and width of the subacromial space), the difference between the extreme values were less than 2 mm, i.e., showing a good consistency. The inter-observer reproducibility was not investigated because the segmentation of the images, in step two of the protocol, is very time-consuming. Consequently, this protocol needs to be enhanced including improved computer-based segmentation of the images.

Thus, before being used in routine practice, this protocol needs to be transferred to an open-bore MRI scanner and needs to be improved by shortening the acquisition times, by visualizing the tendons and by developing a the computer-based segmentation of the images.

## Conclusion

The Dynamic-MRI technique identified kinematic differences between groups of patients with various degrees of cuff tears.

Relative to standard MRI, the Dynamic-MRI has the advantage of providing kinematic data to improve the understanding, diagnosis and best treatment of rotator cuff diseases.

The technique enabled a novel measure; ‘Looseness’, i.e. the translation of the humeral head on the glenoid during an abduction cycle (X_range_ and Y_range_). Looseness was better able at differentiating rotator cuff disease than a simple static measure of relative gleno-humeral position.

The study showed that anterior-posterior gleno-humeral motion increases with rotator cuff disease and it is suggested that a better understanding of this relationship may help improve treatment for rotator cuff diseases.
